# Alterations in integrin expression modulates invasion of pancreatic cancer cells

**DOI:** 10.1186/1756-9966-28-140

**Published:** 2009-10-13

**Authors:** Naomi Walsh, Martin Clynes, John Crown, Norma O'Donovan

**Affiliations:** 1 National Institute for Cellular Biotechnology, Dublin City University, Glasnevin, Dublin 9, Ireland; 2 Dept of Medical Oncology, St Vincent's University Hospital, Dublin 4, Ireland

## Abstract

**Background:**

Factors mediating the invasion of pancreatic cancer cells through the extracellular matrix (ECM) are not fully understood.

**Methods:**

In this study, sub-populations of the human pancreatic cancer cell line, MiaPaCa-2 were established which displayed differences in invasion, adhesion, anoikis, anchorage-independent growth and integrin expression.

**Results:**

Clone #3 displayed higher invasion with less adhesion, while Clone #8 was less invasive with increased adhesion to ECM proteins compared to MiaPaCa-2. Clone #8 was more sensitive to anoikis than Clone #3 and MiaPaCa-2, and displayed low colony-forming efficiency in an anchorage-independent growth assay. Integrins beta 1, alpha 5 and alpha 6 were over-expressed in Clone #8. Using small interfering RNA (siRNA), integrin β1 knockdown in Clone #8 cells increased invasion through matrigel and fibronectin, increased motility, decreased adhesion and anoikis. Integrin alpha 5 and alpha 6 knockdown also resulted in increased motility, invasion through matrigel and decreased adhesion.

**Conclusion:**

Our results suggest that altered expression of integrins interacting with different extracellular matrixes may play a significant role in suppressing the aggressive invasive phenotype. Analysis of these clonal populations of MiaPaCa-2 provides a model for investigations into the invasive properties of pancreatic carcinoma.

## Background

Pancreatic cancer is a devastating disease; it is the eighth most common cause of death (from cancer in both sexes combined) in the World, and is responsible for 227,000 deaths per year [1]. The median survival time after tumour detection is 3-6 months [2], with an all-stage 5-year survival rate of < 5% [3]. Surgery offers the best possibility for survival but at time of diagnosis, only 15% of patients are eligible for resection [4]. The poor outcome is mainly due to difficulties in early detection, lack of an effective treatment and limited understanding of the biological characteristics of this disease. Intrinsic resistance to chemotherapy and radiation [5] coupled with its early systematic dissemination, local tumour progression and metastatic propensity are associated with pancreatic cancer [6].

The processes involved in tumour cell invasion and metastasis are complex. The ability of cancer cells to degrade and adhere to the basement membrane and metastasise to distant organs is one of the most critical aspects of cancer. Adhesion molecules, such as integrins mediate direct cell-cell recognition and cell-matrix interactions [7] are essential for tumour cell migration [8] and for basement membrane penetration [9]. In pancreatic cancer, expression of integrins α6β1 [10-12] and αvβ3 [13] have previously been associated with invasion in cell lines and tissues. However, contrasting results with respect to tumour type and integrin expression patterns makes it difficult to draw general conclusions on the role of specific integrins. Tumour progression and metastasis are associated with changes in a multitude of integrin signalling cascades. Transformed cancer cells are often characterised by the loss/reduction of integrin expression [14,15]. Extracellular matrix (ECM)-ligand binding to an integrin initiates signals, which are transmitted via different, yet interconnecting, pathways and elicit various cell functions, such as morphological changes, adhesion, migration and gene activation, all relevant to the metastatic cascade. The surrounding microenvironment and adhesion properties of pancreatic tumours and sub-populations within the tumour may determine which integrins increase or reduce metastasis in particular tumours [16]. Advanced tumours often contain a variety of sub-populations, which have differing metastatic potential [17]. Li *et al*. [18] identified a highly tumourigenic sub-population of pancreatic cancer cells expressing the cell surface markers CD44, CD24, and epithelial-specific antigen (ESA) capable of self-renewal and increased tumourigenic potential. The identification of pancreatic cancer stem cells has many significant implications for the treatment of pancreatic cancer.

Therefore, in this study, we isolated clonal isogenic sub-populations, derived from the original pancreatic cancer cell line, MiaPaCa-2. Clone #3 and Clone #8 exhibit identical genetic fingerprints with different malignancy-related phenotypes. We examine how altered integrin expression including β1, α5 and α6 affects invasion, motility, adhesion and anoikis using RNAi. Furthermore, the role of integrins in the aggressive invasive phenotype, which correlates with *in vitro *malignant transformation in this pancreatic cancer cell line model, could help to define an invasion/metastatic-related model for pancreatic cancer.

## Methods

### Cell lines

The human pancreatic cell line MiaPaCa-2 was obtained from the European Collection and Cell Cultures (ECACC, UK). Clone #3 and Clone #8 were obtained by limitation dilution cloning in this laboratory, adapted from [19]. The parental cell line was diluted to a concentration of 3 cells/ml and 100 μl plated onto each well of a 96-well plate. After 24 hours each well was studied for single cells, which were allowed to grow into colonies. Once confluence was achieved, cells were transferred to a T25-T75 cm^3 ^flask within 2 weeks. The colonies were then screened by invasion assay to assess their invasive abilities. Cells were maintained in a humidified atmosphere containing 5% CO_2 _at 37°C in Dulbecco's modified Eagles medium (DMEM) supplemented with 5% foetal bovine serum (Sigma-Aldrich). Antibiotics were not used in the growth media. All cell lines were free from Mycoplasma as tested with the indirect Hoechst staining method.

### Invasion and Motility assays

Invasion assays were performed using an adapted method [20]. Matrigel was diluted to 1 mg/ml in serum free DMEM. Laminin, fibronectin and collagen type IV was diluted to 25 μg/ml in PBS and collagen type I to 10 μg/ml. 100 μl of ECM protein was placed into each insert (Falcon) (8.0 μm pore size), in a 24-well plate (Costar). The ECM coated inserts were incubated overnight at 4°C. The following day, the ECM was allowed polymerise at 37°C for 1 hr. The inserts were then washed with serum-free DMEM, 100 μl of complete DMEM was added to the wells and 1 × 10^5^/100 μl cells were then seeded onto the insert. 500 μl of complete DMEM was added into the underside of the well. After 24 hours incubation, the inside of the insert was wiped with a wet cotton swab. The under surface was gently rinsed with PBS and stained with 0.25% crystal violet for 10 minutes, rinsed again with sterile water and allowed to dry. To determine total number of invading cells, the inserts were then viewed under the microscope and the number of cells/field in 10 random fields were counted at 200× magnification. The average number of cells/field was then multiplied by a factor of 140 (growth area of membrane/field area viewed at 200× magnification (calibrated using a microscope graticule)). The mean values were obtained from a minimum of three individual experiments and were subjected to *t*-tests and ANOVA. Motility assays were carried out in the same manner as invasion assays without the addition of ECM on the insert. Experiments were performed in triplicate.

### Adhesion assay

Adhesion assays were performed using a modified method [21]. 24-well plates were coated with 250 μl of 25 μg/ml ECM proteins (laminin, fibronectin and collagen type IV), 10 μg/ml of collagen type I and 1 mg/ml of matrigel. ECM proteins were incubated overnight at 4°C. To reduce non-specific binding, 0.5 ml of 0.1% BSA-PBS solution was added to each well and incubated for 20 minutes, then rinsed twice with sterile PBS. A single cell suspension was obtained, 1 ml of a 2.5 × 10^4 ^cell suspension was added onto the pre-coated 24-well plates in triplicate and allowed to attach for 60 minutes. Blank wells contained ECM proteins but no cells; controls were uncoated wells with cells. After 60 minutes, the non-adhered cells were removed by washing twice with sterile PBS. 200 μl of freshly prepared phosphatase substrate (10 mM *p*-nitrophenol phosphate in 0.1 M sodium acetate, 0.1% Triton X-100 pH 5.5) was added to each well. Plates were then incubated in the dark at 37°C for 2 hours. The enzymatic reaction was stopped by the addition of 100 μl 1 M NaOH. The absorbance was read on a BIO-TEK plate reader at 405 nm with a reference wavelength of 620 nm.

### Anoikis assay

24-well plates were coated with 200 μl of poly-2-hydroxyethyl methacrylate (poly-HEMA, 12 mg/ml dissolved in 95% ethanol, Sigma) and allowed to dry overnight. 1 ml of a single cell suspension of 1 × 10^5^cells was plated onto standard 24 well plates or poly-HEMA coated plates. After 24 hours incubation at 37°C in a humidified atmosphere containing 5% CO_2_, the viability of cells was quantitatively measured using alamarBlue indicator dye (Serotec). The absorbance was read on a BIO-TEC plate reader at 570 nm with a reference wavelength of 600 nm.

### Soft agar colony-forming assay

Soft agar assays or anchorage independent growth assays were carried out using a modified method [22]. 1.548 g of agar (Bacto Difco, 214040) was dissolved in 100 ml of ultra pure water and autoclaved. This agar was then melted in a microwave oven immediately prior to use and incubated at 44°C. 50 ml of agar was then added to 2× DMEM AgarMedium (AgM), mixed well and quickly dispensed onto 35 mm sterile petri dishes. The plates were allowed to set at room temperature and the remaining AgM was returned to the water bath with the temperature reduced to 41°C. 10% FCS was added to the AgM. Cells were harvested and resuspended in medium without serum, ensuring that a single cell suspension was obtained. The cells were diluted to 2 × 10^4 ^cells/ml in a total of 5 ml. 5 ml of agar was then added to each suspension, mixed well and 1.5 ml was dispensed onto each pre-set agar plate, in triplicate, giving a final concentration of 1.5 × 10^4 ^cells per plate. The plates were placed on trays containing a small volume of water to prevent the agar from drying out. On day 0, cells were counted and subsequently cultured for an additional 10 days. After this time the colonies were counted using an inverted microscope at 400×. Ten areas were viewed per plate and the total number of colonies present was extrapolated and the percentage colony forming efficiency (CFE) was determined by expressing the number of colonies formed after 10 days as a percentage of the number of cells counted on day 0.

### Immunoblotting

Whole protein was extracted from cell lysates using 1× lysis buffer (50 mM Tris-Cl, 150 mM NaCl, and 0.5% NP-40). Lysates were centrifuged for 10 min at 14,000 rpm at 4°C. Protein concentrations were determined using the Bio-Rad protein assay according to manufacturer's instructions (Bio-Rad). 35 μg of protein was separated by 7.5% SDS-PAGE under reducing conditions. Proteins were transferred to nitrocellulose membrane (Amersham). Membranes were blocked at 4°C overnight in TBS (25 mM Tris-HCl, pH 7.4, 150 mM NaCl, 2.7 mM KCl) containing 5% (w/v) lowfat milk powder. Membranes were probed with specific antibodies. Anti-β1 (MAB1951Z-20), anti-α5 (AB1949) and anti-α6 (MAB1982) were obtained from Chemicon (Millipore, Europe). Beta-actin was used as loading control (Sigma, A5441). Membranes were washed 3× for 5 min with PBS-Tween-20 (0.1%) and incubated with secondary antibodies, anti-mouse and anti-rabbit (Sigma) for 1 hr at room temperature and washing step repeated. Protein bands were detected with Luminol reagent (Santa Cruz Biotechnology).

### Integrin siRNA transfection

Two integrin β1 (ITGB1) target siRNAs (#109877, #109878 (validated) Ambion Inc.) were used to silence integrin β1 expression. Two integrin α5 (ITGA5) target siRNAs (#106728, #111113 Ambion Inc.) and two integrin α6 (ITGA6) target siRNAs (#8146, #103827 (validated) Ambion Inc.) were used to silence the respective target genes. Solutions of siRNA at a final concentration of 30 nM were prepared in OptiMEM (Gibco™). NeoFX solution was prepared in OptiMEM and incubated at room temperature for 10 min. After incubation, an equal volume of neoFX solution was added to each siRNA solution, mixed well and incubated for a further 10 min. 100 μl of neoFX/OptiMEM solutions were added into a 6 well plate in duplicate. Clone #8 (3 × 10^5^) cells were added onto the siRNA solution. The plates were gently mixed and incubated for 24 hours. The transfection mixture was removed and replaced with fresh medium. Positive control, kinesin (Ambion Inc.) was included in each triplicate experiment. Invasion, motility, adhesion and anoikis assays were then carried out 48 hours after transfection, as previously described.

### Statistical analysis

Student's t-test was used for statistical analyses of invasion, motility, adhesion, anoikis and soft agar assays. * p ≤ 0.05, ** p ≤ 0.01, *** p ≤ 0.005 indicated statistical significance. Data are presented as mean ± standard deviation. Each experiment was repeated at least three times. Multiple group comparison experiments were validated by ANOVA.

## Results

### Single cell cloning

**Figure 1:**
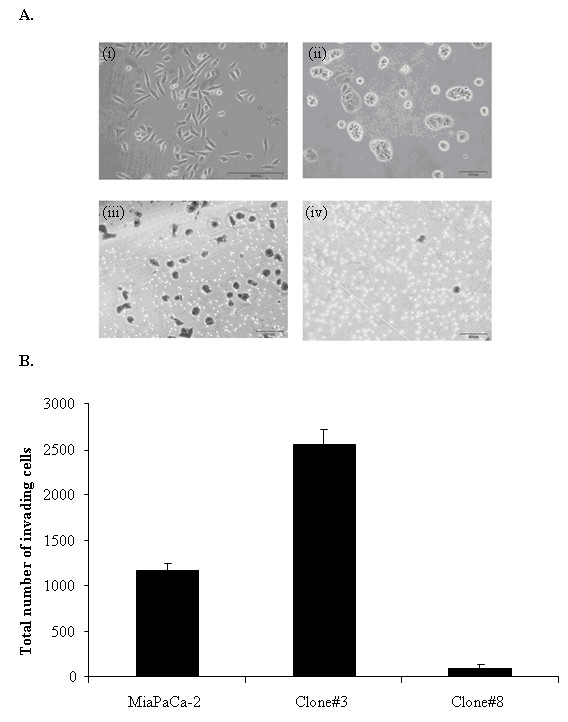
**A. Morphology of the highly invasive (i) Clone #3 with elongated and spindle-like phenotype and low-invasive (ii) Clone #8 with epithelial tight colonies**. Cell invasion assay representing (iii) Clone #3 and (iv) Clone #8 invading through ECM coated Boyden chamber, stained with crystal violet. Magnification 200×. Scale bar, 200 μm. B. Total number of invading cells. Results shown are a minimum of three repeats ± standard deviation (*n *= 3).

Four clones were isolated from the pancreatic cell line, MiaPaCa-2 and successfully established as cell lines. The invasion status of the clones was tested using the Boyden chamber assay with inserts coated with matrigel. Two sub-populations, Clone #3 and Clone #8, showed a significant increase (Clone #3, 2.5-fold increase, *p *= 0.001) and decrease (Clone #8, 12-fold decrease, *p *= 0.00001), ANOVA (*p *< 0.001), (Fig 1A(i-ii) and 1B) in invasion through matrigel, compared to the parental MiaPaCa-2 cells. These two clonal populations also displayed distinct morphological differences (Fig 1A(iii-iv)). The invasive cell line, Clone #3 displayed an elongated spindled shaped morphology, similar to mesenchymal cells. Clone #8, low invasion, was similar to epithelial cells in tight clustered colonies.

### Invasion and adhesion to ECM proteins

**Figure 2:**
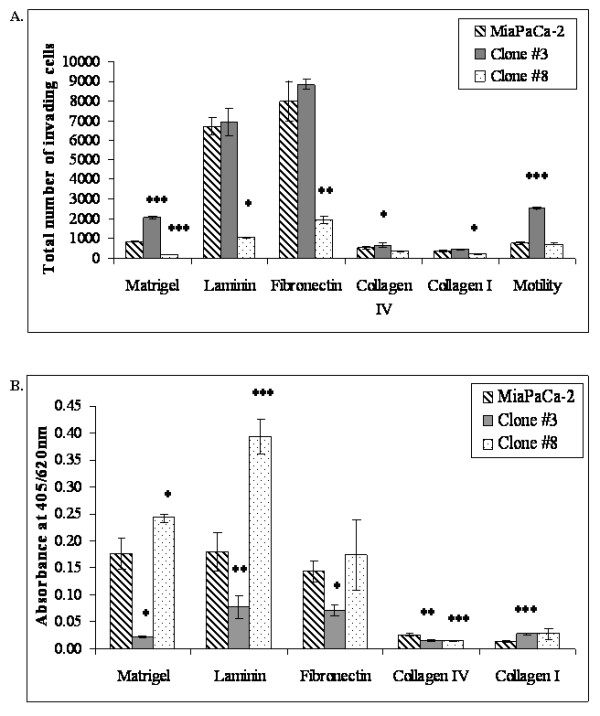
**A. Invasion assay of MiaPaCa-2, Clone #3 and Clone #8 through ECM proteins**. Motility assay refers to invasion assay without the presence of ECM. Results are displayed as the total mean number of cells invading at 200× magnification (*n *= 3). B. Adhesion of MiaPaCa-2, Clone #3 and Clone #8 to ECM proteins: matrigel, laminin, fibronectin, collagen type IV (ANOVA, all p < 0.001) and collagen I (ANOVA p = 0.04). Results are expressed as absorbance at 405 nm with a reference wavelength of 620 nm. Data shown is mean ± standard deviation (*n *= 3). Student's *t*-test; *p *≤ 0.05*, 0.01**, 0.005***.

Invasion of MiaPaCa-2 and sub-populations, Clone #3 and Clone #8, through a range of ECM proteins was examined (Fig 2A). The invasion of MiaPaCa-2 and Clone #3 is comparable through laminin and fibronectin whereas Clone #8 showed a significant decrease in invasion, 6.3 and 4.0-fold (*p *= 0.002, *p *= 0.008) through laminin and fibronectin, respectively, ANOVA (all *p *< 0.001). Low invasion was observed for Clone #3 through collagens type I and IV; Clone #8 showed significantly decreased invasion through the collagens (1.6 and 1.6-fold (*p *= 0.03, *p *= 0.02)), ANOVA (*p *= 0.007, *p *= 0.001). Interestingly, the lowest level of invasion displayed by the cell lines was through the collagens, type IV and I, which is in agreement with previous studies indicating MiaPaCa-2 does not express collagen-binding integrins [23]. The highest level of invasion was observed through fibronectin. Clone #3 also displayed significantly increased motility (*p *= 0.00005) whereas the motility of Clone #8 was similar to that of MiaPaCa-2, ANOVA (*p *< 0.001) (Fig 2A).

The more invasive Clone #3, displays significantly decreased adhesion to matrigel (*p *= 0.01), laminin (*p *= 0.02), fibronectin (*p *= 0.01) and collagen type IV (*p *= 0.01) compared to the parental cell line (Fig 2B). In contrast a significant increase in adhesion was observed to collagen type I (*p *= 0.003), although the level of adhesion to the collagens was significantly lower than that to fibronectin or laminin. The less invasive Clone #8, showed significantly increased adhesion to matrigel (*p *= 0.04) and laminin (*p *= 0.002). Adhesion to fibronectin and collagen type I were also increased, but not significantly and adhesion to collagen type IV was decreased significantly (*p *= 0.001) for Clone #8.

### Anoikis and anchorage-independent growth

**Figure 3:**
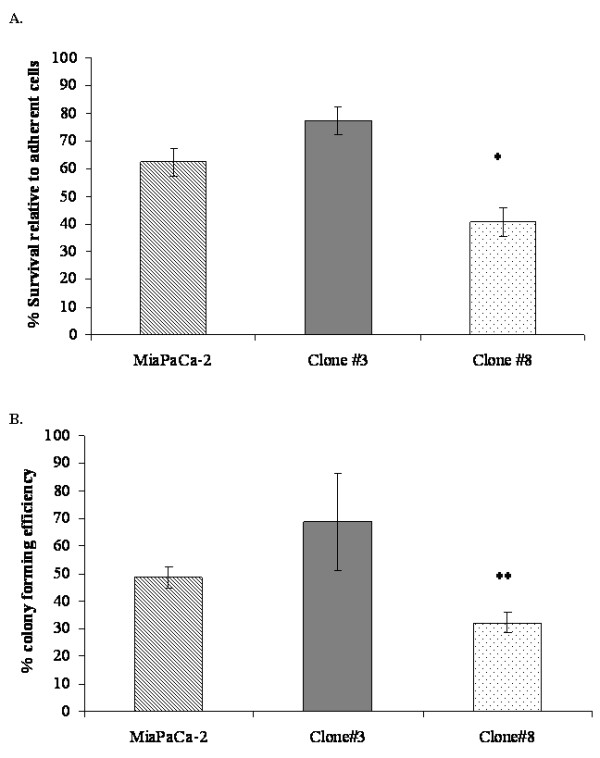
**A. Percentage survival of MiaPaCa-2, Clones #3 and Clone #8 in suspension compared to adherent cells, ANOVA (*p *= 0.002)**. B. Percentage colony formation efficiency (%CFE) of MiaPaCa-2, Clone #3 and Clone #8 under anchorage-independent growth conditions, ANOVA (*p *= 0.02). Data shown is mean ± standard deviation (*n *= 3). Student's *t*-test; *p *≤ 0.05*, 0.01**, 0.005***.

The evaluation of survival in suspension (anoikis) showed that Clone #3 was resistant to anoikis compared to the parental cell line, although this difference did not reach statistical significance (*p *= 0.07). Clone #8 demonstrated a significant sensitivity to anoikis (*p *= 0.02) compared to the parental cell line, MiaPaCa-2 (Fig 3A). Anchorage-independent growth was assessed using the soft agar assay. MiaPaCa-2 showed colony formation with an average colony size of 75 μm and percentage colony forming efficiency (% CFE) of 48%; Clone #3 formed more and larger colonies with an average size of 120 μm and a %CFE of 69%. In contrast, Clone #8 (low invasion and high adhesion), showed significantly reduced ability (32% CFE) to form colonies (*p *= 0.006) and the average size of colonies was 60 μm (Fig 3B).

### Integrin expression

**Figure 4:**
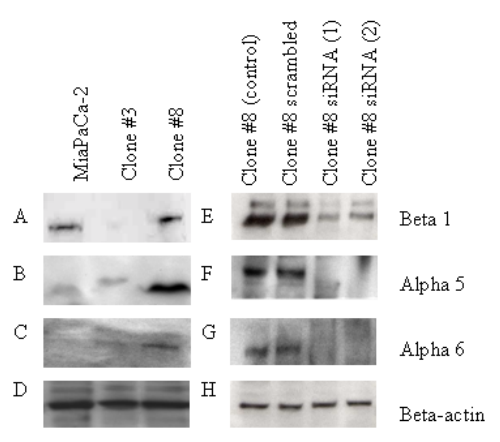
**Immunoblot of A. Integrin β1 B. Integrin α5 C. Integrin α6 and D. β-actin used as loading control in MiaPaCa-2, Clone #3 and Clone #8**. E. Knockdown of integrin β1 in Clone #8 cells 48 hours post transfection (siRNAs ITGβ1 #1 and #2). F. Knockdown of integrin α5 in Clone #8 cells 48 hours post transfection (siRNAs ITGα5 #1, #2). G. Knockdown of integrin α6 in Clone #8 cells 48 hours post transfection (siRNAs ITGα6 #1 and #2). H. Beta-actin used as loading control.

Significant changes in invasion and adhesion to fibronectin and laminin were observed in the sub-populations. Therefore, expression of integrins β1, α5 and α6, which are associated with adhesion to laminin and fibronectin were examined in the cell lines, by immunoblotting (Fig 4A-C). Beta-actin used as loading control (Fig 4D). Compared to MiaPaCa-2, Clone #8 showed higher expression of integrins β1 and α5. Low levels of α6 were detected in Clone #8, while it was undetectable in the parental MiaPaCa-2 cells. Lower levels of each of the integrins were detected in Clone #3 compared to Clone #8.

### Integrin β1 knockdown

**Figure 5:**
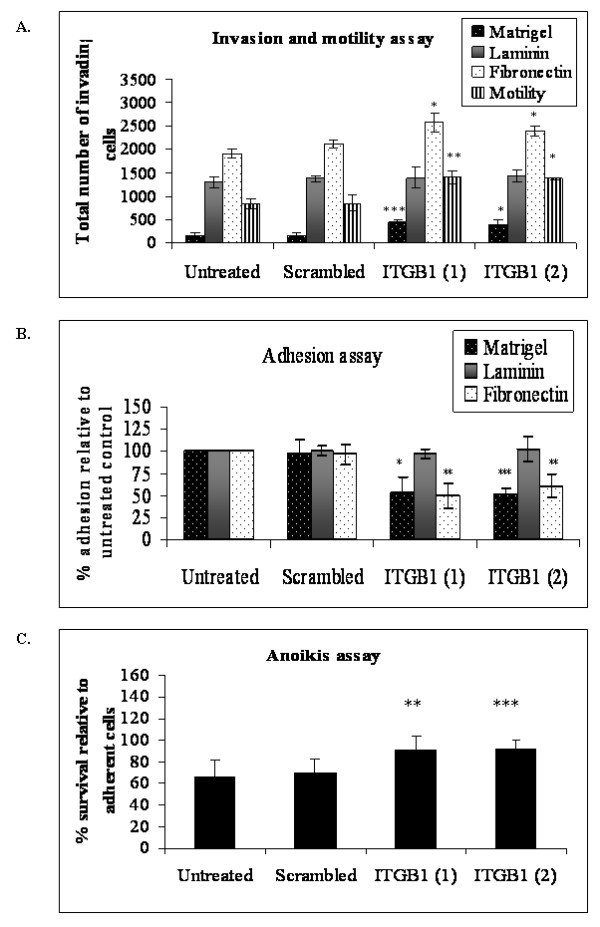
**A. Invasion of Clone #8 through matrigel, laminin and fibronectin and motility assay**. B. Adhesion assay of Clone #8 to matrigel, laminin and fibronectin. C. Anoikis assay. Experiments were performed 48 hours post-transfection with two different exon targeted siRNA integrin Beta 1. Student's *t*-test; *p *≤ 0.05*, 0.01**, 0.005***.

The role of integrin β1 in the low invasive cell line, Clone #8 was investigated using RNAi. Clone #8 was chosen as it expresses high levels of integrin β1 compared to Clone #3 (Fig 4A). Cells were subjected to invasion, motility, adhesion and anoikis assays following siRNA transfection. SiRNA knockdown of protein was confirmed by immunoblot (Fig 4E). Integrin β1 siRNA transfected into Clone #8 resulted in a significant increase in invasion through matrigel (*p *= 0.005 and *p *= 0.04), ANOVA (*p *= 0.006), although invasion through laminin was not significantly altered. Invasion through fibronectin was significantly increased (*p *= 0.04 and *p *= 0.02), ANOVA (*p *= 0.02). Motility of Clone #8 after siRNA β1 transfection was also significantly increased (*p *= 0.01 and *p *= 0.03) compared to the scrambled control, ANOVA (p = 0.003) (Fig 5A). A significant decrease in adhesion to matrigel (45-47%) was observed (*p *= 0.02 and *p *= 0.002), ANOVA (*p *= 0.002), while adhesion to fibronectin (*p *= 0.02 and *p *= 0.04), ANOVA (*p *= 0.01) was significantly decreased with the integrin β1 siRNA treatment (Fig 5B). Adhesion to laminin was not altered after transfection with integrin β1 siRNAs. Anoikis assays were also carried out to investigate whether the knockdown of integrin β1 had any effect on the survival of Clone #8 in suspension (Fig 5C). A significant increase in the percentage of cells surviving in suspension was observed after treatment with integrin β1 siRNA compared to cells treated with scrambled control (*p *= 0.01, *p *= 0.003), ANOVA (*p *= 0.005)

### Integrin α5 and α6 knockdown

**Figure 6:**
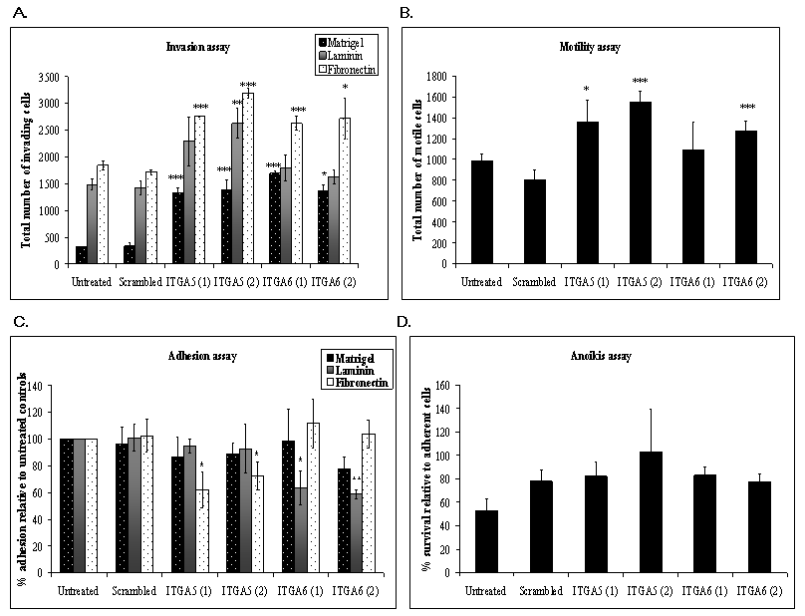
**A. Invasion through matrigel, laminin and fibronectin**. B. Motility assay. C. Adhesion assay to matrigel, laminin and fibronectin. D. Anoikis assay of Clone #8 control, treated with scrambled siRNA, two independent integrin ITGα5 siRNA targets and two integrin ITGα6 target siRNAs. Student's *t*-test; *p *≤ 0.05*, 0.01**, 0.005***.

To further evaluate the role of specific integrins in invasion, motility, adhesion and anoikis, siRNA experiments targeting α5 and α6 integrins were also carried out in Clone #8 cells (Fig 4F-G). Transfection of integrin α5 siRNA into Clone #8 resulted in an increase in invasion through matrigel (*p *= 0.0003, *p *= 0.005), ANOVA (*p *< 0.001) laminin (*p *= 0.07, *p *= 0.008), ANOVA (*p *= 0.001) and fibronectin (*p *= 0.0002, *p *= 0.0001), ANOVA (*p *< 0.001) compared to the scrambled control. Transfection of siRNA α6 into Clone #8 resulted in a significant increase in invasion through matrigel (*p *= 0.00009 and *p *= 0.02), ANOVA (*p *< 0.001) and fibronectin (*p *= 0.004 and *p *= 0.04), ANOVA (*p *= 0.04), with no significant increase in invasion through laminin (Fig 6A). Knockdown of integrin α5 resulted in significantly increased motility, ANOVA (*p *= 0.007) while integrin α6 knockdown also increased motility significantly in one siRNA (*p *= 0.19 and *p *= 0.004), ANOVA (*p *= 0.04) (Fig 6B).

A slight decrease in adhesion to matrigel and laminin was observed although not significantly, while a significant reduction in adhesion to fibronectin was observed after integrin α5 siRNA treatment of Clone #8 cells (*p *= 0.02, *p *= 0.03), ANOVA (*p *= 0.02). Adhesion to matrigel and fibronectin was not altered with integrin α6 siRNA treatment; however adhesion to laminin was reduced (*p *= 0.08 and *p *= 0.01), ANOVA (*p *= 0.01) (Fig 6C). No significant change in anoikis response was observed after either integrin α5 and α6 siRNA transfection, compared to cells treated with scrambled control (Fig 6D).

## Discussion

One of the most lethal aspects of pancreatic cancer is its early systemic dissemination and tumour progression [24]. The inability to diagnose pancreatic cancer at an early stage has contributed to poor prognosis, as well as the difficulties in treating the metastatic disease. The exact mechanism of pancreatic invasion and metastasis has not been fully elucidated and a better understanding of these processes is essential in treating this disease.

To study the inherent heterogeneity of differing sub-populations within a tumour, we isolated isogenic clonal populations from the human pancreatic cell line, MiaPaCa-2, by single cell cloning. Two sub-populations displaying differences in invasion were further analysed to characterise the *in vitro *invasive phenotype. Clone #3 was characterised as highly invasive and motile with decreased adhesion to ECM proteins. The less invasive Clone #8 displayed increased adhesion to ECM proteins. Neither clone showed an affinity to collagen type I and IV. Grzesiak *et al*. [23] previously determined that the parental cell line MiaPaCa-2 does not express collagen-binding integrins α1 and α2, but showed that the cells are metastatic in an orthotopic mouse model and preferentially migrate on laminin-1. Although collagen type IV constitutes the major intrinsic component of the extracellular matrix [25], the ability of the clonal populations in our study to invade or/adhere to matrigel could be due to laminin, another major component of the ECM, and to a lesser extent fibronectin, which represents a significant step in metastasis [26]. Changes in adhesive characteristics, invasion and motility of cells have been suspected to play a role in mediating the spread of malignant cells.

Clone #3 displays the characteristics of an aggressive cancer, with decreased adhesion facilitating increased motility and invasion, coupled with the ability to survive and to form colonies in anchorage independent conditions. These features could be compared to the *in vivo *situation where the ability of tumour cells to detach from the primary tumour, invade through the ECM, survive in the blood stream, and invade and form tumours at secondary sites, leads to the formation of metastases. Therefore, we believe that Clone #3 represents an *in vitro *model of tumour cells with increased metastatic potential. In contrast Clone #8 appears to be a model of tumour cells with decreased metastatic potential, showing decreased invasion, increased adhesion, increased sensitivity to anoikis and reduced ability to grow and form colonies in anchorage-independent conditions.

Integrins are involved in regulating growth, differentiation, and death by regulating the interaction between cell and ECM [7]. In pancreatic cancer, links have previously been established between increased invasion and decreased adhesion to ECM proteins *in vitro *and to high metastatic potential *in vivo *[27-29].

In general, the loss or gain of expression of individual integrins appears to be indirectly associated with malignant transformation and involved in tumour progression and metastasis. Over expression of α5β1 in CHO cells demonstrated reduced malignancy [30], whereas α2β1 and α3β1 were expressed in non-neoplastic and fibroadenomas but were low or absent in highly invasive mammary carcinomas [31]. In our study, Clone #3 showed reduced expression of integrins β1, α5 and α6 compared to Clone #8, which correlates with the reduced adhesion to laminin and fibronectin, as integrin α5β1 is a receptor for fibronectin and α6β1 is a receptor for laminin [32,26]. Integrin β1, α5 and α6 siRNA transfection in Clone #8 resulted in significantly increased motility and invasion through matrigel and fibronectin, and reduced adhesion to matrigel and fibronectin. Loss of integrin β1 did not alter the invasion or adhesion of Clone #8 cells to laminin, but loss of α6 significantly reduced adhesion to laminin. These results suggest that inhibition of integrin β1 alone is not sufficient to block adhesion to laminin. Other integrin complexes such as α6β4 [33] could control laminin-mediated adhesion/invasion in these cells. Gilcrease *et al*. [34] showed that α6β4 cross linking in suspended non adherent breast cancer cells resulted in cell surface clustering of EGFR, increasing EGFR-mediated activation of Rho in response to EGF, which may lead to tumour cell migration. Knockdown of the expression of integrin β1 in Clone #8 also revealed a more anoikis resistant phenotype. Disruption of β1 integrin complexes has previously implicated in induction of anoikis [35-37]. These experiments support the hypothesis that decreased expression of these integrins β1, α5 and α6, in Clone #3 plays a role in the aggressive invasive phenotype observed *in vitro*. Reduced expression of integrin β1, but not α5 and α6, appears to play an important role in anoikis resistance in this model. Therefore, targeting of integrins specific to certain tumours may provide viable options for therapeutic treatment.

## Conclusion

We have established that sub-populations within a pancreatic cancer cell line display varied invasion and adhesive interactions with ECM proteins. Low adhesion, high motility and invasion, reduced integrin α5, α6 and β1 expression, anoikis resistance and anchorage-independent growth in Clone #3 represents a highly invasive phenotype. This is the first study to report the relationship between invasion, adhesion, anoikis and anchorage independent colony formation within sub-populations of a pancreatic cancer cell line. *In vivo *analysis of these clonal populations of MiaPaCa-2 will be required to determine if the aggressive invasive phenotype *in vitro *correlates with increased metastatic potential *in vivo*. Further investigation of this aggressive phenotype may help to identify novel markers and targets for invasion and metastasis in pancreatic cancer.

## Competing interests

The authors declare that they have no competing interests.

## Authors' contributions

NW carried out all experimental analysis, participated in design of the study and drafted the manuscript. MC and NOD conceived of the study, and participated in its design and coordination and helped to draft the manuscript. JC contributed to the design of the study. All authors read and approved the final manuscript.
